# Low-Temperature Sintering of Bi(Ni_0.5_Ti_0.5_)O_3_-BiFeO_3_-Pb(Zr_0.5_Ti_0.5_)O_3_ Ceramics and Their Performance

**DOI:** 10.3390/ma16093459

**Published:** 2023-04-28

**Authors:** Wuyang Wang, Shihao Wang, Jun Sun, Qiushi Wang, Bijun Fang

**Affiliations:** 1 Bell Honors School, Nanjing University of Posts and Telecommunications, Nanjing 210023, China; q20010103@njupt.edu.cn; 2 School of Materials Science and Engineering, Jiangsu Collaborative Innovation Center of Photovolatic Science and Engineering, Jiangsu Province Cultivation Base for State Key Laboratory of Photovoltaic Science and Technology, National Experimental Demonstration Center for Materials Science and Engineering, Changzhou University, Changzhou 213164, China; a815305547@163.com (S.W.); s21010856082@smail.cczu.edu.cn (J.S.); s21010856057@smail.cczu.edu.cn (Q.W.)

**Keywords:** Bi(Ni_0.5_Ti_0.5_)O_3_ end component, sintering aid, sintering temperature, relaxation behavior, electrical performance

## Abstract

A low-temperature sintering strategy was realized for preparing 0.21Bi(Ni_0.5_Ti_0.5_)O_3_-0.05BiFeO_3_-0.74Pb(Zr_0.5_Ti_0.5_)O_3_ (0.21BNT-0.05BF-0.74PZT) ceramics by conventional ceramic processing by adding low melting point BiFeO_3_ and additional sintering aid LiBO_2_. Pure perovskite 0.21BNT-0.05BF-0.74PZT ceramics are prepared at relatively low sintering temperatures, and their structure presents tetragonal distortion that is affected slightly by the sintering temperature. The 1030 °C sintered samples have high densification accompanied by relatively large grains. All ceramics have excellent dielectric performance with a relatively high temperature of dielectric constant maximum, and present an apparent relaxation characteristic. A narrow sintering temperature range exists in the 0.21BNT-0.05BF-0.74PZT system, and the 1030 °C sintered 0.21BNT-0.05BF-0.74PZT ceramics exhibit overall excellent electrical performance. The high-temperature conductivity can be attributed to the oxygen vacancies’ conduction produced by the evaporation of Pb and Bi during sintering revealed by energy dispersive X-ray measurement.

## 1. Introduction

Due to the rapid development of science and technology, high-Curie-temperature (T_C_) piezoelectric materials are required urgently because of the severe and harsh operation conditions in the application fields such as metallurgy, petroleum exploration, aeronautics and astronautics, and so on [1]. As piezoelectric ceramics are used extensively world-wide, Pb(Zr_1−x_Ti_x_)O_3_-based (PZT) ceramics cannot meet such strict application requirements since the commercially used PZT5H ceramics have just a typical T_C_ of 193 °C [2]. The T_C_ temperature is rather low for application in such circumstances since piezoelectric materials are faced with an obsession of depolarization and the safe working temperature is normally far less than their T_C_ temperature [3,4]. Therefore, developing high-T_C_ piezoelectric materials with a large energy density to replace the PZT-based piezoceramics has become an increasingly researched hotspot in recent years [5].

As well as consideration of the operating temperature, designing the composition is very important since electrical performance is determined mainly by the composition of the piezoelectric materials. Discovering the morphotropic phase boundary (MPB) is still an important strategy to develop novel piezoelectric materials since PZT exhibits enhanced electrical performance with composition approaching the MPB [6], which is also applicable to other ferroelectric systems [7]. The improved phase structure and physical properties are normally correlated with the coexistence of multiferroelectric phases, and further study has discovered intermediate bridge ferroelectric phases with lower symmetry [8,9].

Among versatile ferroelectrics, a LiNbO_3_ single crystal has a high T_C_ of 1210 °C, but its piezoelectricity is too poor and crystal growth is complex and expensive. BiScO_3_ ceramics have a high production cost, and a Bi(Ni_1/2_Ti_1/2_)O_3_-based system has a difficult preparation process as discussed in our previous research [10]. The combination of Bi and (Ni_1/2_Ti_1/2_) induces our interest. Bi(Me)O_3_, where Me can be a single trivalent ion such as In, Yb, Sc, etc., can also be our considered complex ions combination such as Ni_1/3_Nb_2/3_, Zn_1/2_Ti_1/2_, Mg_1/2_Zr_1/2_, etc., and its complex perovskite solid solution with PbTiO_3_ was reported by Eitiel et al. [11]. Such a result promoted the research of similar systems, and MPB composition and excellent piezoelectricity in binary Bi(Ni_1/2_Zr_1/2_)O_3_-PbTiO_3_ and Bi(Ni_0.5_Ti_0.5_)O_3_-PbTiO_3_ were reported thereafter [12,13]. Then, ternary system BiScO_3_-Bi(Ni_1/2_Zr_1/2_)O_3_-PbTiO_3_ was reported by Zhao et al., which presents a rather large piezoelectric constant d_33_ of 480 pC/N and a high T_C_ of 439 °C [14].

Based on these research studies, a pseudo-ternary system was constructed in this work, where Bi(Ni_0.5_Ti_0.5_)O_3_ (BNT) was selected as one end component, which has a perovskite structure with a B-site occupied by equivalent Ni^2+^ and Ti^4+^ ions [15], and Pb(Zr_0.5_Ti_0.5_)O_3_ was selected as another end component, which can stabilize the perovskite structure more effectively than PbTiO_3_. Ji et al. devised a pseudo-binary system xBi(Ni_1/2_Ti_1/2_)O_3_-(1−x)Pb(Zr_1/2_Ti_1/2_)O_3_ (xBNT-(1−x)PZT), in which preparation feasibility and MPB-dependent electrical properties were studied, and the 0.25BNT-0.75PZT ceramics presented excellent performance with d_33_ = 510 pC/N and T_C_ = 227 °C [16], consistent with the design of this composition. The third end component selected was BiFeO_3_ (BF), substituting out BiScO_3_ due to the consideration of raw material cost. Furthermore, BF presents multiferroic performance with a high T_C_ of 810 °C, which has large theoretical saturation polarization but cannot normally be acquired due to preparation difficulty and large leakage conductivity [17]. Although pure BF ceramics were hard to prepare, BF could facilitate ceramics preparation, promote grain growth and increase densification process due to its low melting point of 930 °C, and it had many application instances [18,19]. A similar sintering aid characteristic is used to facilitate the preparation of Bi(Ni_0.5_Ti_0.5_)O_3_-BiFeO_3_-Pb(Zr_0.5_Ti_0.5_)O_3_ (BNT-BF-PZT) ceramics in this work.

In materials science, composition, structure, performance and application have complicated mutual interactions [20], among which composition designing and ceramics processing are very important influencing factors. In this work, 0.21Bi(Ni_0.5_Ti_0.5_)O_3_-0.05BiFeO_3_-0.74Pb(Zr_0.5_Ti_0.5_)O_3_ (0.21BNT-0.05BF-0.74PZT) was chosen due to the comprehensive consideration of its performance and preparation feasibility based on the analysis of the reported ternary phase diagram and preliminary experimental study [13,16,21]. Among ceramics processing, sintering conditions are the most important factors since the phase structure, point defects, grain size, density, etc., are affected greatly by the sintering temperature and soaking time, especially for the difficult to prepare Pb- and Bi-containing ceramics [22]. Therefore, a low-temperature sintering strategy was realized due to the low melting point of BF [19] and PbO accompanied by the adding of an additional sintering aid, LiBO_2_ [23], and the systematic influences on structure, performance and conduction mechanism were studied in detail.

## 2. Experimental Procedure

0.21Bi(Ni_0.5_Ti_0.5_)O_3_-0.05BiFeO_3_-0.74Pb(Zr_0.5_Ti_0.5_)O_3_ (0.21BNT-0.05BF-0.74PZT) ceramics were prepared by conventional ceramic processing via low-temperature sintering technique with additional adding 0.5 wt% LiBO_2_ as sintering aid. Well-mixed stoichiometrically weighted raw materials of Bi_2_O_3_ (>99%), NiO (>99%), TiO_2_ (>98.5%), Fe_2_O_3_ (>99.9%), PbO (>99%) and ZrO_2_ (>99%) were calcined at 850 °C for 2 h to obtain perovskite structure 0.21BNT-0.05BF-0.74PZT precursor powder. Sintering aid was added into the well-ground precursor powder, then, pelletization with addition of 5 wt% polyvinyl alcohol aqueous solution and formed by cold-pressing at 300 MPa were undertaken. After separate debinding at 550 °C for 30 min, the green discs were sintered between 1010 °C and 1050 °C with 10 °C temperature interval covered by the calcined precursor powder. Detailed preparation procedure can be seen elsewhere for preparing the 0.55Bi(Ni_0.5_Ti_0.5_)O_3_-0.05BiFeO_3_-0.4PbTiO_3_ system [21].

Phase structure evolution of the polished 0.21BNT-0.05BF-0.74PZT ceramics was characterized by Rigaku D/max-2500/PC X-ray Diffractometer (XRD, Tokyo, Japan) in continuous scanning mode, where the measurement 2θ range was 10–80°, step interval was 0.02° and scanning speed was 4°/min. Ceramics’ free surface morphology was observed by JEOL JSM-IT100 InTouchScope^TM^ scanning electron microscope (SEM, Tokyo, Japan) using 800 °C thermal etched 30 min samples. Silver electrode was formed by firing silver paste at 810 °C for 10 min for electrical properties’ measurement. The dielectric performance–temperature relationship and impedance spectra at elevated temperatures were measured by Partulab HDMS-1000 connected with Microtest 6630-10 Precision LCR Meter (Wuhan, China). Piezoelectric constant and electromechanical coupling performance were measured by ZJ-3AN quasi-static d_33_ tester (Beijing, China) and TH2826 impedance analyzer (Changzhou, China), respectively, using room-temperature poled samples in silicone oil for 1 min [21].

## 3. Results and Discussion

### 3.1. Structure and Density Characterization

For the design of the composition in this work, as well as the consideration of MPB based on the phase diagram and preliminary experiment, Goldschmidt’s tolerance factor *t* is an important technique to check perovskite structural distortion and the stability of the ABO_3_ complex oxides as shown below [24]:

$$t=\frac{r_{A}+r_{O}}{\sqrt{2}\left(r_{B}+r_{O}\right)}$$

Using the revised effective ionic radii calculated by Shannon [25], the tolerance factor *t* can be calculated, where the ionic radii of Pb^2+^ in 12-CN (coordination number) is 1.49 Å, Ni^2+^ (6-CN) 0.69 Å, Ti^4+^ (6-CN) 0.605 Å, Fe^3+^ (6-CN, high spin state) 0.645 Å, Zr^4+^ (6-CN) 0.72 Å and O^2-^ (6-CN) 1.4 Å. The dilemma is the ionic radius for Bi^3+^ just having maximum 8-CN with 1.17 Å. Via linear extrapolation of the ionic radius based on the relationship between the ionic radii and the coordination number, the predicted ionic radius for Bi^3+^ with 12-CN would be 2.06 Å. Then, the calculated *t* value range is 0.9643–1.0438 with Bi^3+^ in 8-CN to 12-CN. As the calculated *t* value approaches 1, it reveals the perovskite structure stability of the designed 0.21BNT-0.05BF-0.74PZT composition [24].

**Figure 1:**
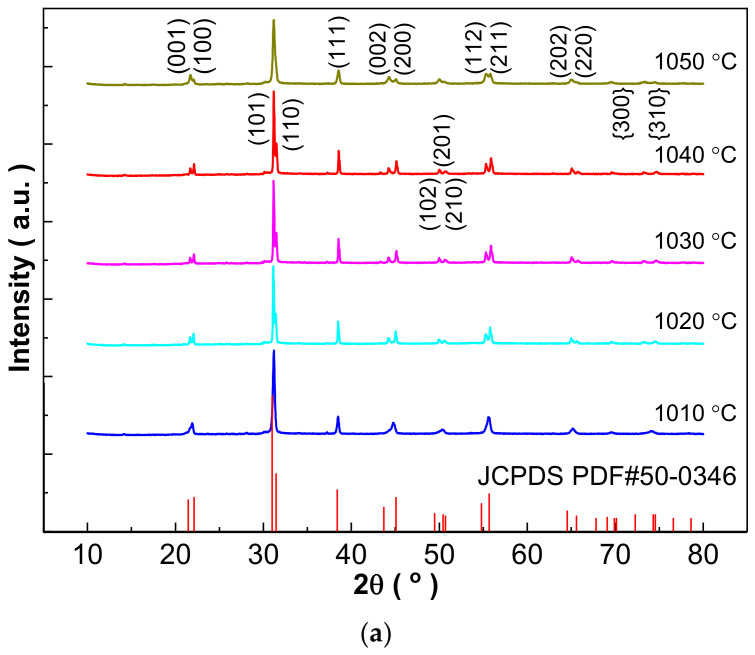

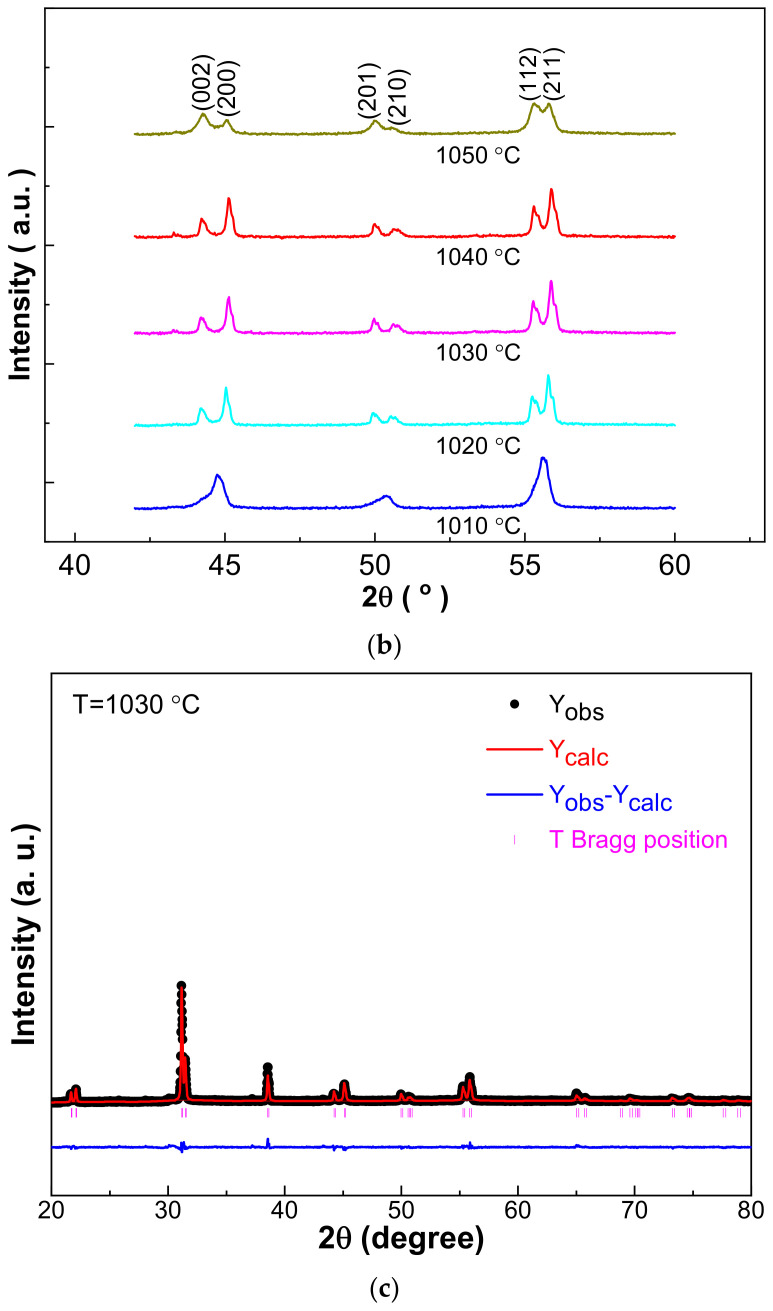
(**a**) XRD patterns of low-temperature sintered 0.21BNT-0.05BF-0.74PZT ceramics sintered between 1010 °C and 1050 °C; (**b**) zoomed XRD patterns with 2θ being 42–60°; (**c**) Rietveld refinement of 1030 °C sintered 0.21BNT-0.05BF-0.74PZT ceramics.

The successful design of the composition is further confirmed by XRD measurement as shown in Figure 1. A pure perovskite phase is obtained for all the 0.21BNT-0.05BF-0.74PZT ceramics sintered at relatively low sintering temperatures, and no impurity is detected within the XRD measurement limitation. In the enlarged XRD patterns shown in Figure 1b, it can be clearly seen that the phase structure evolution is induced by increasing the sintering temperature. Within the 2θ degree range of 42–60°, the {200}, {210} and {211} diffraction reflections show broadened singlet-like peaks for the 1010 °C sintered sample, showing a typical MPB composition characteristic [26]. For the 1020–1050 °C sintered ceramics, apparent splitting appears in the above three diffraction peaks accompanied by the change in diffraction peak intensity of the split peaks. The apparent splitting of the {211} diffraction reflection reveals the existence of rhombohedral distortion [26].

Since the major phase shows tetragonal symmetry, the phase structure of the 0.21BNT-0.05BF-0.74PZT ceramics is refined by the Rietveld method and indexed as a tetragonal phase (referred to JCPDS 50-0346). Figure 1c shows XRD Rietveld refinement using the 1030 °C sintered 0.21BNT-0.05BF-0.74PZT ceramics as an example. Based on this, lattice parameters were calculated and are shown in Table 1 combined with refinement parameters. All ceramics have excellent fitting accuracy since all fitting parameters are less than R_wp_ ≤ 6.12%, R_p_ ≤ 4.25%, and χ^2^ ≤ 5.770, showing high reliability of the Rietveld method. Table 2 shows density data, where theoretical density was calculated based on the XRD calculated cell volume and chemical formula weight, and bulk density was measured by Archimedes’ water immersion method [21]. The low-temperature sintering technique with the addition of LiBO_2_ is successful in this work since a large density can be obtained. Within the narrow sintering temperature range, the primary cell volume and relative density present a similar change trend, i.e., increasing first and then decreasing with an increasing sintering temperature. The best ceramics are prepared at a rather low sintering temperature of 1030 °C, and their density is 7.802 g/cm^3^ and relative density reaches 96.56%, satisfying the production and quality requirements of the electronic ceramic industry. The tetragonality factor c/a also exhibits an increasing first and then decreasing variation affected by sintering temperature, showing the change characteristic of tetragonal distortion [27].

### 3.2. SEM Observation

**Figure 2:**
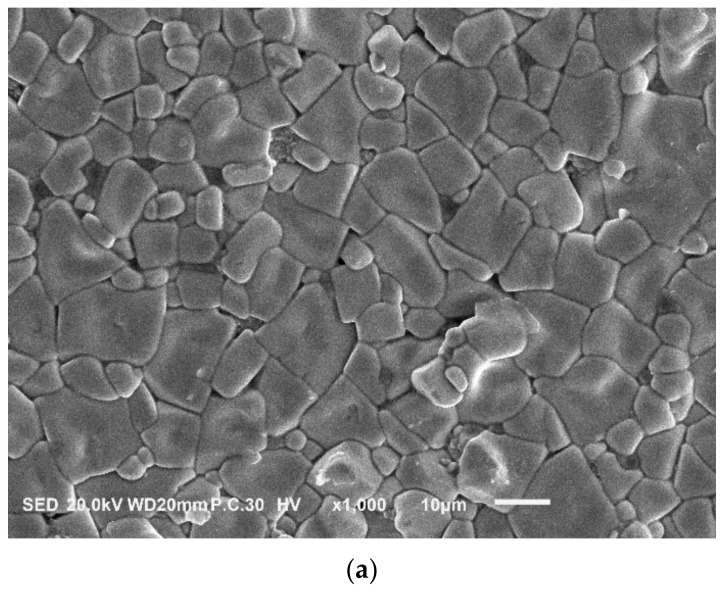

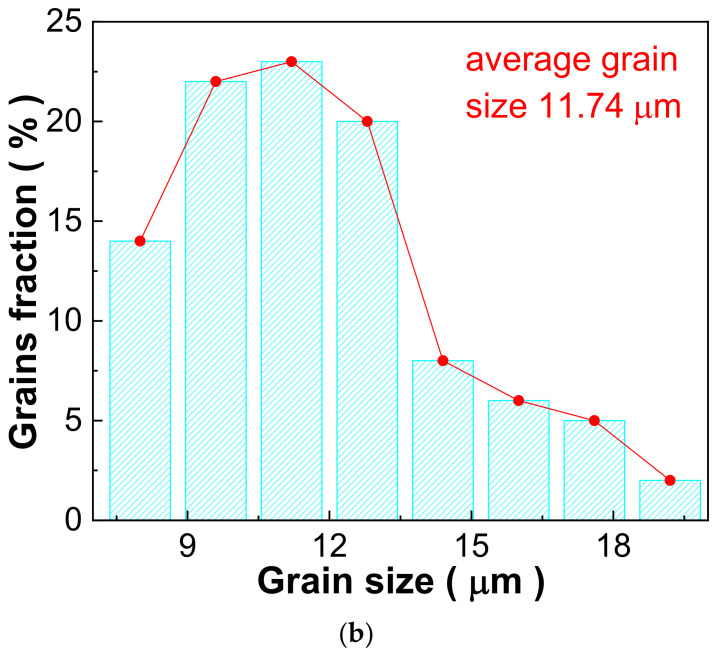
SEM image (**a**) and corresponding grain size distribution statistics based on two micrographs (**b**) of 1030 °C sintered 0.21BNT-0.05BF-0.74PZT ceramics.

The largest densification of the 0.21BNT-0.05BF-0.74PZT ceramics sintered at 1030 °C is confirmed in Figure 2 by SEM observation accompanied by the corresponding grain size statistics. The free surface shows an obvious grain boundary, with clear grains and almost without visible pores, consistent with the high density. For the low-temperature sintered ceramics, no other characteristic grains appear, further showing the stability of the perovskite structure obtained in this work. Due to the low-temperature sintering technology, the solid-state sintering mechanism has a major effect in the ceramics’ densification process since most grains have a polyhedron shape with a large aspect ratio. The liquid phase sintering mechanism also contributes to the sintering process due to the low melting point of Pb and Bi oxides [18,19], and it produces relatively large grains with an average grain size of 11.74 μm. Due to the utilization of the low-temperature sintering technique, a large grain size appears in the 0.21BNT-0.05BF-0.74PZT ferroelectric ceramics, which can improve the ceramics’ quality and generate an extrinsic contribution to electrical performance [28].

### 3.3. Resistance and Dielectric Performance

From Table 2 it can be seen that the resistivity of all of the low-temperature sintered 0.21BNT-0.05BF-0.74PZT ceramics is larger than 10^11^ Ω·cm, which is beneficial as it increases the dielectric breakdown strength and obtains large piezoelectricity due to increasing the poling electric field [29]. The resistivity versus the sintering temperature relationship also presents the largest value at the 1030 °C sintering temperature, which can be attributed to the largest relative density.

**Figure 3:**
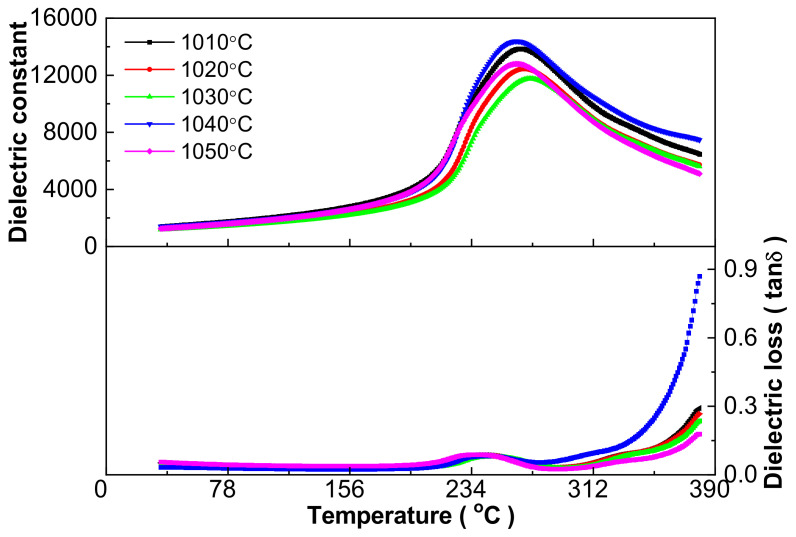
Dielectric performance versus temperature relationship at 1 kHz of 0.21BNT-0.05BF-0.74PZT ceramics sintered between 1010 and 1050 °C.

The influence of sintering temperature on the dielectric performance of the 0.21BNT-0.05BF-0.74PZT ceramics is shown in Figure 3. All curves present a similar shape, where broad dielectric constant-temperature curves accompanied by a sole wide dielectric peak can be attributed to the relaxor ferroelectric state to paraelectric phase transition. Important dielectric performance parameters are given in Table 3. With the increase in sintering temperature, the dielectric constant peak temperature (T_m_) increases first and reaches a maximum value of 269 °C at the 1030 °C sintering temperature, and then declines with a variation of 10 °C in T_m_, whereas the dielectric constant maximum value (ε_m_) decreases first, then increases to a maximum value of 16,085.5 at the 1040 °C sintering temperature, and finally decreases again with a variation of 3409.6. Both maximum values appear at different sintering temperatures, which is also considered to relate to the relaxor ferroelectric characteristic [8].

**Figure 4:**
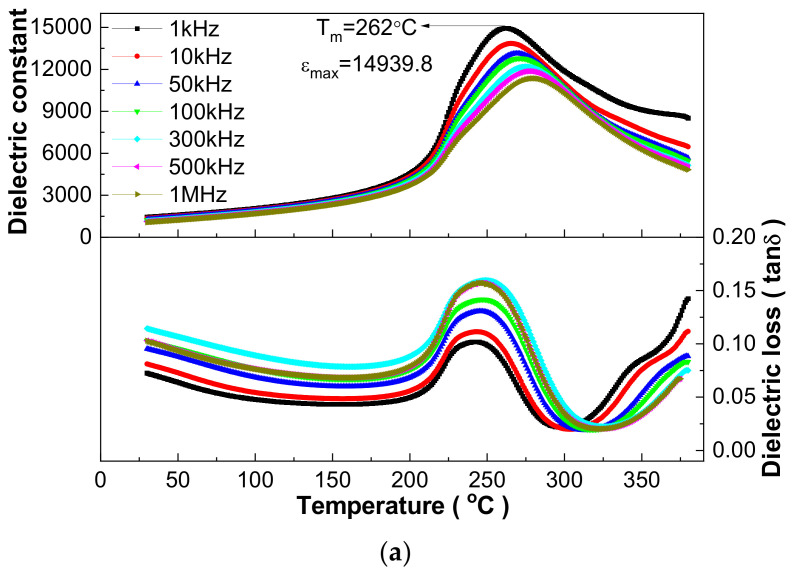

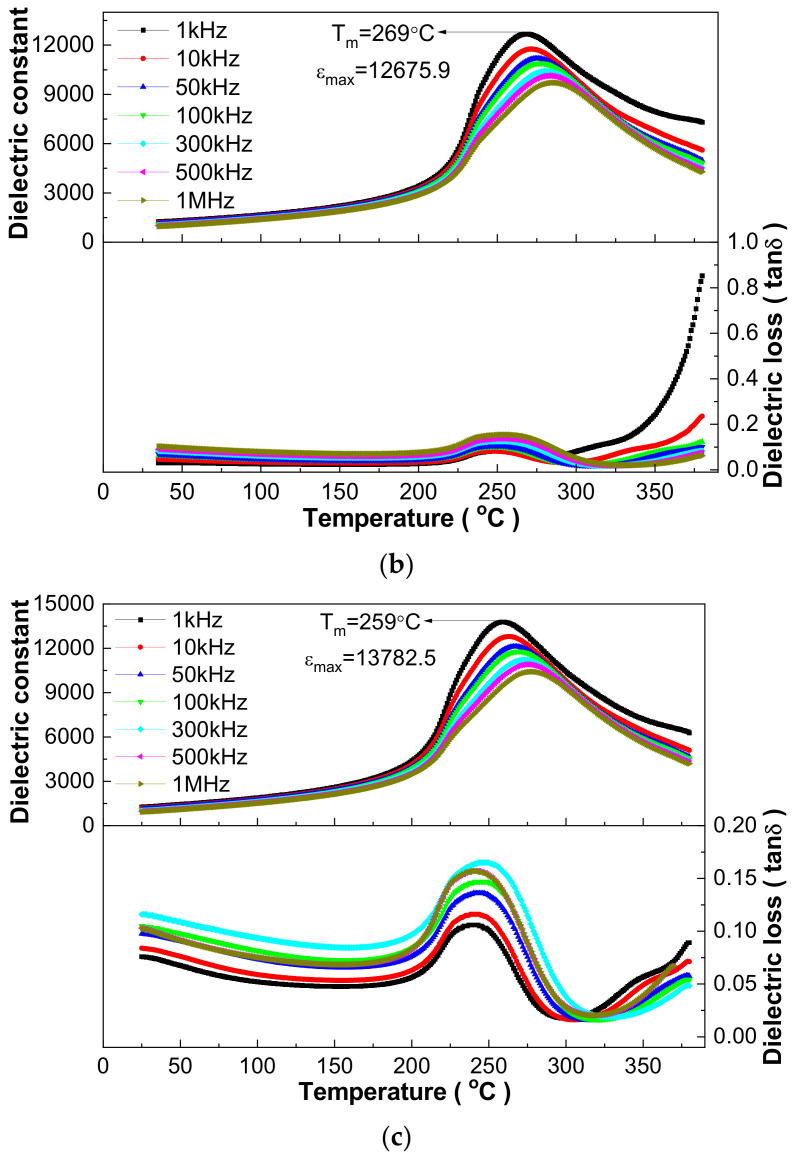
Typical dielectric performance–temperature curves measured at several frequencies upon heating of 0.21BNT-0.05BF-0.74PZT ceramics sintered at (**a**) 1010 °C; (**b**) 1030 °C and (**c**) 1050 °C.

The relaxor behavior is further shown with dielectric performance at different frequencies in Figure 4 using three samples as examples. Actually, all samples have apparent dielectric frequency dispersion, broad dielectric peaks and a diffused phase transition characteristic, i.e., presenting an obvious difference in the T_m_ and ε_m_ values at different frequencies, although detailed values show a difference for the ceramics sintered at different temperatures. Normally, dielectric loss exhibits a more apparent frequency dispersion, and the temperature of the loss tangent (tanδ) extremum value (T_m_’) is lower than T_m_ for relaxor ferroelectrics [8]. The difference in the above two temperatures (T_m_ and T_m_’) can be regarded as a signature of the degree of relaxor behavior. The unusual fast increase in dielectric loss above the T_m_’ temperature (Figure 3 and Figure 4) can be attributed to the evaporation of Pb and Bi during sintering, although using the low-temperature sintering technique, which generates point defects, causes thermally activated lossy conduction and leads to an abnormal increase in dielectric loss [30] as shown below [29]:

(1) $$Pb_{Pb}^{\times }+O_{O}^{\times }\to Pb↑+\frac{1}{2}O_{2}↑+V_{Pb}^{\prime \prime }+V_{O}^{\cdot \cdot }$$

(2) $$2Bi_{Bi}^{\times }+3O_{O}^{\times }\to 2Bi↑+\frac{3}{2}O_{2}↑+2V_{Bi}^{\prime \prime \prime }+3V_{O}^{\cdot \cdot }$$

**Figure 5:**
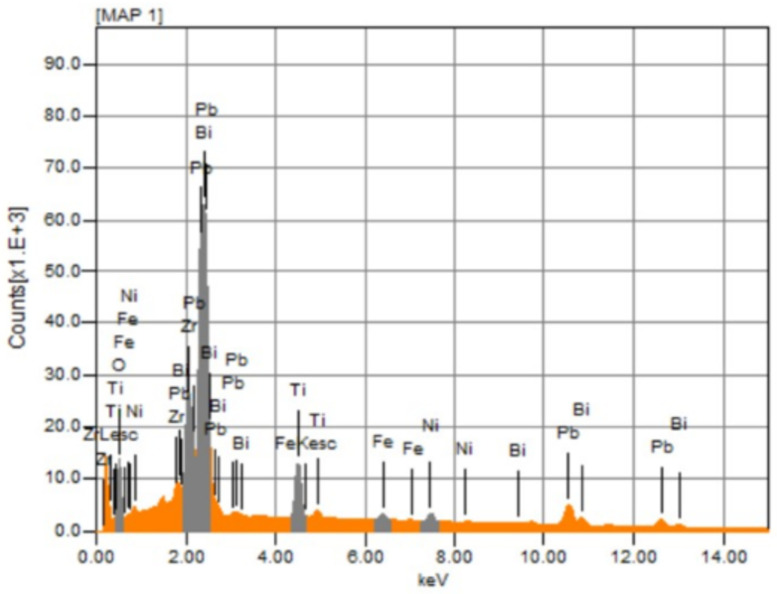
Typical EDX spectrum of 1030 °C sintered 0.21BNT-0.05BF-0.74PZT ceramics.

The evaporation of Pb and Bi was detected by energy dispersive X-ray (EDX) measurement as shown in Figure 5, Figures S1 and S2 and Table 4. Since the free surface of ceramics was used for the EDX measurement and the sample was thermally etched at 800 °C for 30 min, the atomic percentage of all metal elements was far less than the designed composition. Such a difference can be used to reveal the evaporation of metal elements, especially for Bi and Pb, during the sintering process. Although the evaporation of some metals during sintering is inevitable, the distribution of metal elements is rather homogeneous as shown in Figure S2, where the grain boundary presents higher sublimation capability due to the amorphous state or disordered arrangement of atoms [19].

**Figure 6:**
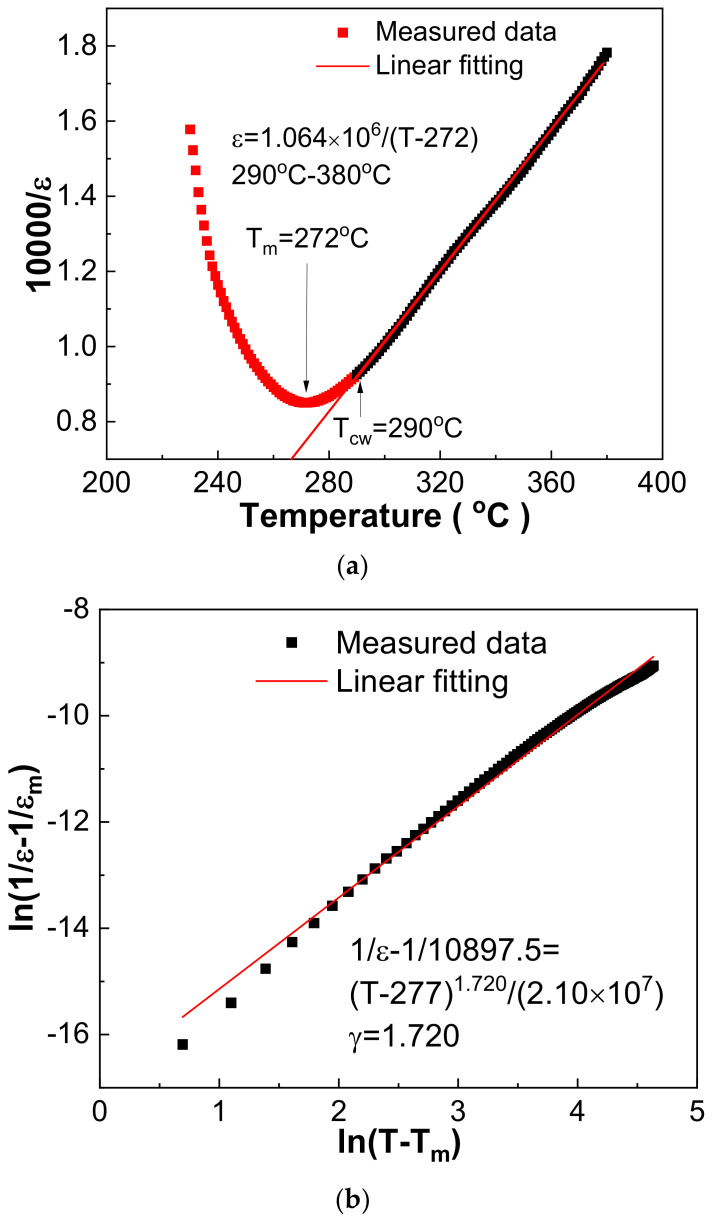
Dielectric response fitting of 1030 °C sintered 0.21BNT-0.05BF-0.74PZT ceramics using 10 kHz data. (**a**) Curie–Weiss law; (**b**) quadratic law.

Dielectric response fitting using dielectric data above the T_m_ temperature provides an effective way to discriminate ferroelectrics’ type. Using the 1030 °C sintered 0.21BNT-0.05BF-0.74PZT ceramics as an example, the fitted Curie–Weiss law is ε = 1.064 × 10^6^/(T − 272) and the fitted exponential law is 1/ε − 1/10897.5 = (T − 277)^1.720^/(2.10 × 10^7^) (Figure 6) [31,32]. Combined with the data shown in Table 3, the fitted Curie–Weiss constant C of all samples has 10^5^ magnitude order. Moreover, the C value presents a decreasing trend, the Curie–Weiss temperature increases first and then decreases, and the temperature above which the Curie–Weiss law can be obeyed (T_CW_) shows an increasing trend with increasing sintering temperature, i.e., the temperature difference of T_CW_-T_m_ is 11–27 °C for different samples. The fitted diffused coefficient γ increases first and reaches a maximum value of 1.733 for the 1040 °C sintered 0.21BNT-0.05BF-0.74PZT ceramics, and then decreases slightly, revealing the change in the relaxation characteristic degree. All the fitted γ values are larger than 1.69 but less than 2. Based on these results, the 0.21BNT-0.05BF-0.74PZT samples present complicated dielectric response behavior, and can be regarded as displacive driven ferroelectric phase transition superimposed on obvious frequency dispersion [32]. Such a phase transition characteristic is normally related to the generation of polar nano-regions (PNRs) caused by local nano-sized nonuniformity of chemical composition, disordered distribution of cations at the A-site and B-site of the perovskite structure [33], and a randomly distributed strain field and electric field [34].

**Figure 7:**
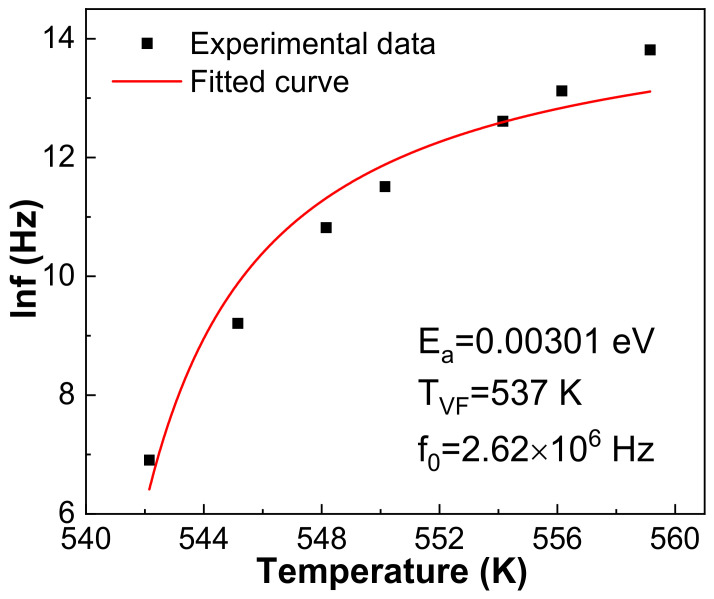
Vogel–Fulcher fitting of 1030 °C sintered 0.21BNT-0.05BF-0.74PZT sample.

Vogel–Fulcher fitting provides another technique to research relaxation characteristic as shown in Figure 7, also using the 1030 °C sintered 0.21BNT-0.05BF-0.74PZT ceramics as an example. Based on the Vogel–Fulcher formula, f = f_0_exp[E_a_/k_B_(T − T_VF_)], the fitting uses 1–1000 kHz data; therefore, the empirical relaxation strength ΔT_res_ = T_m(1000 kHz)_ − T_m(1 kHz)_ can be used to describe the frequency dispersion degree [34]. The fitted parameters are f_0_ = 2.62 × 10^6^ Hz, E_a_ = 0.00301 eV, T_VF_ = 537 K and ΔT_res_ = 17 K, respectively, presenting the appearance of composition-induced relaxor-like ferroelectrics. The average activation energy E_a_ = 0.00301 eV is comparable with the thermal excitation energy of kT, reflecting that the dipoles’ polarization orientation relates to the thermal excitation process [34]. The fitted Debye frequency f_0_ = 2.62 × 10^6^ Hz is comparable to that of the Ba(Zr_0.30_Ti_0.70_)O_3_ ceramics, correlating with the complex microstructure and the generated point defects due to the evaporation of Pb and Bi during sintering [34].

### 3.4. Piezoelectric Performance

**Figure 8:**
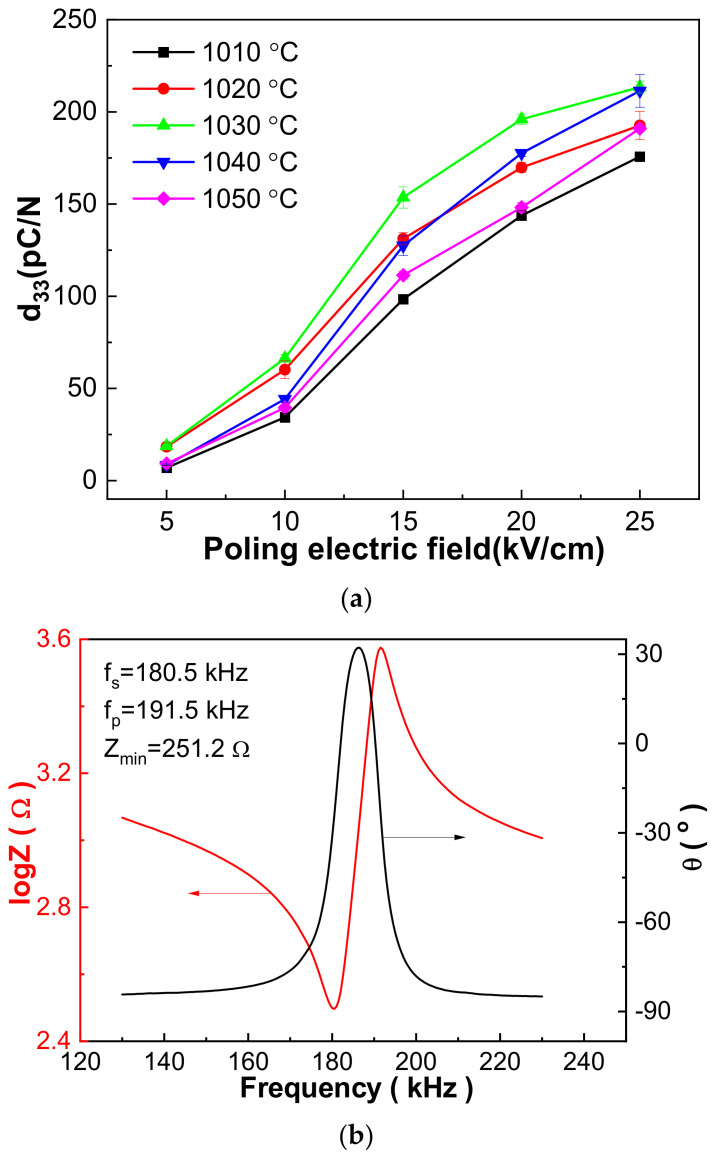
(**a**) Poling electric-field-dependent piezoelectricity d_33_ of 0.21BNT-0.05BF-0.74PZT samples sintered at different temperatures; (**b**) impedance and phase degree versus frequency curves of 1030 °C sintered 0.21BNT-0.05BF-0.74PZT ceramics poled at 25 kV/cm.

Figure 8 shows the piezoelectric performance of the 0.21BNT-0.05BF-0.74PZT samples. With the increase in the poling electric field, piezoelectric constant d_33_ increases rapidly, correlating with the easy switching of 180° domains under a high external electric field [5]. With the elevation of the sintering temperature, the d_33_ value increases first and then decreases. The 1030 °C sintered 0.21BNT-0.05BF-0.74PZT ceramics have the largest d_33_ value, being 213.4 pC/N, which is rather large considering the high T_m_ temperature. The 1030 °C sintered 0.21BNT-0.05BF-0.74PZT ceramics present explicit resonant and anti-resonant shape behavior poled at 25 kV/cm, whereas the maximum phase degree θ is just 34°, showing the possibility of increasing the poling electric field but constrained by the leakage current of the samples. Such a low θ value accompanied from the beginning by a rather low minus θ degree, i.e., nearly −90°, is usually observed in the (K_1−x_Na_x_)NbO_3_-based lead-free piezoelectric ceramics, and the reason is difficult to explain [35]. Based on the resonant spectra, the mechanical quality factor Q_m_ and electromechanical coupling coefficient K_p_ were calculated and are shown in Table 5. The 0.21BNT-0.05BF-0.74PZT ceramics exhibit an apparent soft piezoelectric materials characteristic due to the existence of a large quantity of point defects as discussed in defect Equations (1) and (2). Overall, the 1030 °C sintered 0.21BNT-0.05BF-0.74PZT ceramics have excellent electrical properties, where d_33_ = 213.4 pC/N, K_p_ = 37.7% and Q_m_ = 27.3.

### 3.5. Conduction Mechanism

The impedance spectra and conduction mechanism were studied using the 1030 °C sintered 0.21BNT-0.05BF-0.74PZT ceramics as an example. The real part of impedance Z′ tends to decrease sharply before a certain frequency and then becomes stable, and the imaginary part of impedance Z″ normally presents a peak with the increase in frequency. With the increase in measurement temperature, both the Z′ and Z″ impedance values decrease, the Z″ peak frequency moves to a high frequency and the Z′’ peak broadens obviously.

**Figure 9:**
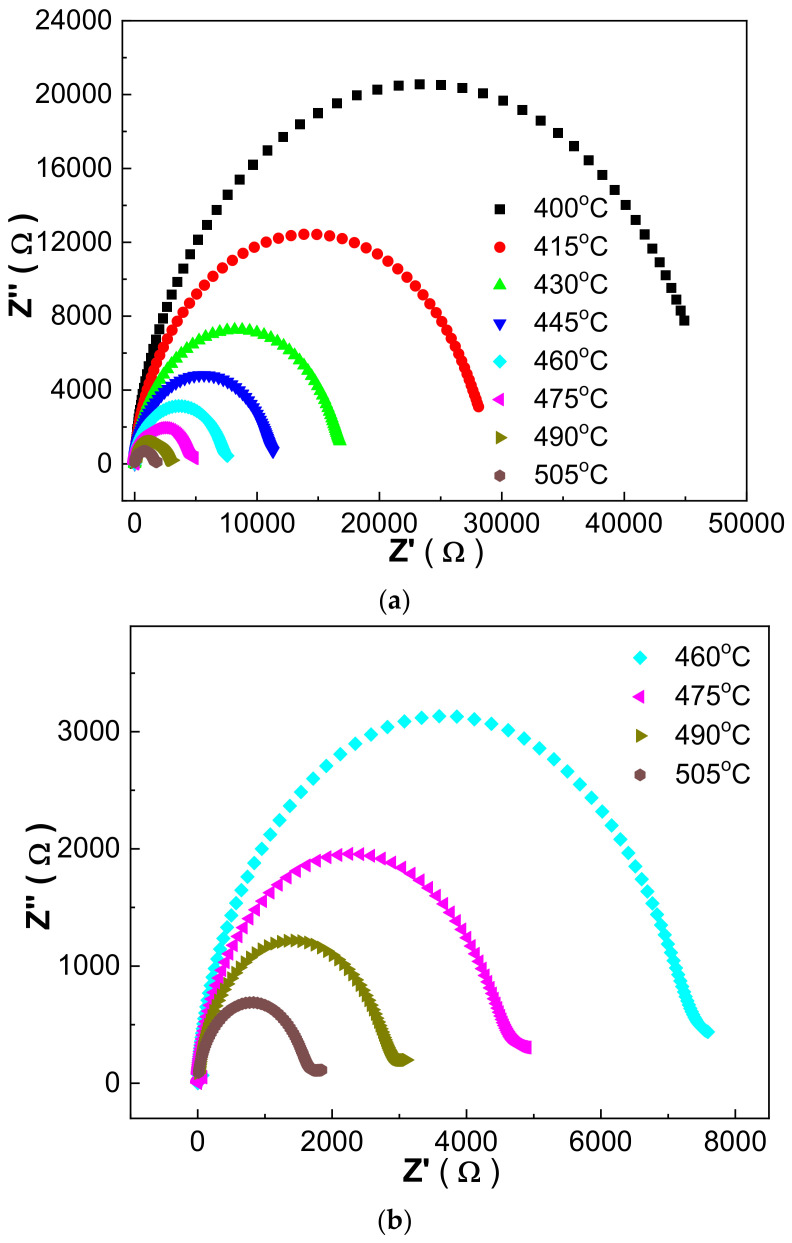
(**a**) Cole–Cole impedance spectra of 1030 °C sintered 0.21BNT-0.05BF-0.74PZT ceramics measured within 400–505 °C; (**b**) enlarged Cole–Cole impedance spectra measured within 460–505 °C.

Figure 9 shows Cole–Cole-like Z′-Z″ relationship curves based on the impedance measurement. At lower temperatures, the curves exhibit a straight line shape, whereas perfect Cole–Cole-shaped semi-circles are obtained at higher temperatures. The curve at different temperatures just presents a sole semi-circle, revealing the contribution of the grains’ conduction, and correlating with the thermally activated conduction of point defects generated as shown in defect Equations (1) and (2) [36].

**Figure 10:**
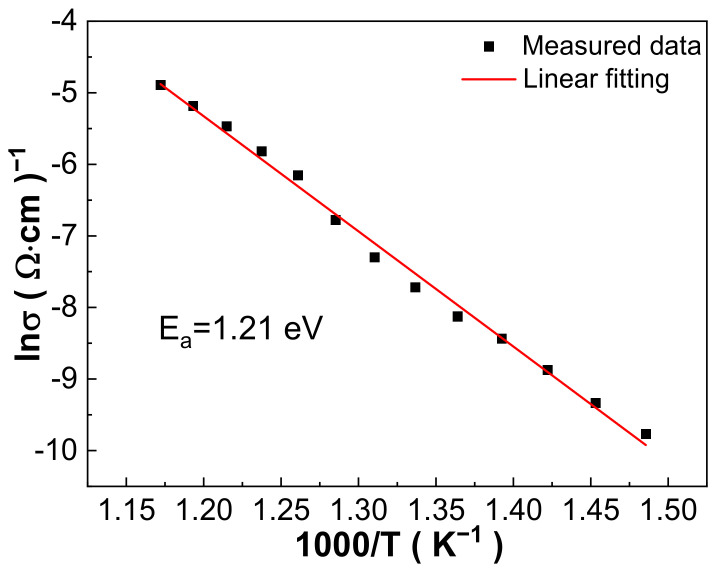
Conductivity–temperature relationship of 1030 °C sintered 0.21BNT-0.05BF-0.74PZT ceramics.

To reveal the type of point defects, the high-temperature conductivity was linearly fitting according to the Arrhenius law, based on which, the activation energy E_a_ of the sample was calculated and the point defects’ type could be determined. The sample’s conductivity was extrapolated from impedance spectra via formula σ = t/RS, in which t is the thickness of the sample, S is the sample’s area and R is the real part of the impedance at low frequencies. The calculated conductivity and liner fitting result are shown in Figure 10, where the fitting line matches well with the extrapolated data and shows the reliability of the Arrhenius law fitting. The fitted E_a_ = 1.21 eV, approaching close to 1 eV, shows that the oxygen vacancies produced by the evaporation of Pb and Bi during sintering as discussed in defect Equations (1) and (2) dominate the conduction process in the 0.21BNT-0.05BF-0.74PZT ceramics at high temperatures [37].

## 4. Conclusions

Pure perovskite 0.21BNT-0.05BF-0.74PZT ceramics were prepared by low-temperature sintering technology, which presents a narrow sintering temperature range, and the tetragonal distortion c/a increases first and then decreases with the increasing sintering temperature. Within the narrow sintering temperature range, the primary cell volume and relative density present parabolic curve-like change, and the best ceramics are prepared at a low sintering temperature of 1030 °C accompanied by large densification of 96.56% and rather uniform micromorphology. All of the sintered 0.21BNT-0.05BF-0.74PZT ceramics have resistivity larger than 10^11^ Ω·cm and excellent dielectric properties, where the highest T_m_ 269 °C and largest ε_m_ 16,085.5 are acquired at 1030 °C and 1040 °C sintering temperatures, respectively. The relaxation characteristic is confirmed by exponential law dielectric response fitting and Vogel–Fulcher fitting combined with dielectric frequency dispersion and diffused phase transition. The 1030 °C sintered 0.21BNT-0.05BF-0.74PZT ceramics have excellent electrical properties, in which T_m_ = 269 °C, d_33_ = 213.4 pC/N, K_p_ = 37.7% and Q_m_ = 27.3. Oxygen vacancies produced due to the evaporation of Bi and Pb during sintering revealed by the EDX measurement perform a control function in the high-temperature grains’ conduction. The rather large piezoelectricity combined with a relatively high T_m_ temperature provides the prospect of their application in harsh operation temperature fields for the 0.21BNT-0.05BF-0.74PZT ceramics studied.

## Figures and Tables

**Table 1:** Lattice constants with P4mm space group and fitting precision parameters of 0.21BNT-0.05BF-0.74PZT ceramics sintered at different temperatures refined by Rietveld method.

Sintering Temperature (°C)	a = b (Å)	c (Å)	α = β = γ (°)	Reliability Factors
1010	4.046819	4.071851	90	R_wp_ = 6.12%, R_p_ = 4.25%, χ^2^ = 5.770
1020	4.024786	4.091517	90	R_wp_ = 5.56%, R_p_ = 3.94%, χ^2^ = 4.492
1030	4.015606	4.091098	90	R_wp_ = 4.84%, R_p_ = 3.51%, χ^2^ = 3.587
1040	4.016916	4.090731	90	R_wp_ = 4.82%, R_p_ = 3.54%, χ^2^ = 3.700
1050	4.030802	4.094163	90	R_wp_ = 4.16%, R_p_ = 3.11%, χ^2^ = 2.607

**Table 2:** Cell volume, density and resistance of low-temperature sintered 0.21BNT-0.05BF-0.74PZT ceramics at several sintering temperatures.

Sintering Temperature (°C)	Cell Volume (Å^3^)	Bulk Density (g/cm^3^)	Theoretical Density (g/cm^3^)	Relative Density (%)	Resistivity (Ω·cm)
1010	66.68366	7.242	8.005032	90.46	3.56 × 10^11^
1020	66.27808	7.677	8.043474	95.45	4.21 × 10^11^
1030	65.96933	7.802	8.078878	96.56	6.68 × 10^11^
1040	66.00646	7.550	8.080214	93.45	1.22 × 10^11^
1050	66.51936	7.258	8.016112	90.54	1.74 × 10^11^

**Table 3:** Important dielectric parameters of 0.21BNT-0.05BF-0.74PZT ceramics sintered at several temperatures.

Sintering Temperature (°C)	Dielectric Peak Temperature T_m_ (°C)	Maximum Dielectric Constant (ε_m_)	Curie–Weiss Constant (C)	Curie–Weiss Temperature (°C)	T_CW_ (Obey Curie–Weiss Law Temperature) (°C)	Diffuseness Coefficient (γ)
1010	262	14,939.8	1.277 × 10^6^	266	277	1.697
1020	265	13,435.5	1.075 × 10^6^	268	281	1.705
1030	269	12,675.9	1.064 × 10^6^	272	290	1.720
1040	260	16,085.5	1.064 × 10^6^	266	290	1.733
1050	259	13,782.5	8.849 × 10^5^	263	290	1.698

**Table 4:** Element type and corresponding atomic percentage of 1030 °C sintered 0.21BNT-0.05BF-0.74PZT ceramics measured at different locations.

Element	Atom% (Location 1)	Atom% (Location 2)	Atom% (Location 3)
Bi	8.73 ± 0.07	8.82 ± 0.07	9.53 ± 0.07
Ni	3.26 ± 0.02	2.18 ± 0.02	3.47 ± 0.02
Ti	11.22 ± 0.01	11.31 ± 0.01	11.94 ± 0.02
Fe	1.90 ± 0.01	1.70 ± 0.01	2.07 ± 0.01
Pb	19.05 ± 0.07	19.27 ± 0.07	19.76 ± 0.07
Zr	8.42 ± 0.03	9.19 ± 0.03	8.51 ± 0.03
O	47.42 ± 0.02	47.53 ± 0.02	44.71 ± 0.02

**Table 5:** K_p_ and Q_m_ values of 0.21BNT-0.05BF-0.74PZT ceramics sintered at different temperatures poled under 25 kV/cm.

Sintering Temperature (°C)	K_p_ (%)	Q_m_
1010	30.4	13.3
1020	30.5	15.4
1030	37.7	27.3
1040	32.3	14.3
1050	37.3	19.5

## Data Availability

All data that support the findings of this study are included within the article and Supplementary File.

## Supplementary Materials

The following supporting information can be downloaded at: https://www.mdpi.com/article/10.3390/ma16093459/s1, Figure S1: EDX spectra of 1030 °C sintered 0.21BNT-0.05BF-0.74PZT ceramics at different locations; Figure S2: Element surface mapping of 1030 °C sintered 0.21BNT-0.05BF-0.74PZT ceramics at different locations.
