# Supplementary material for: Low-Temperature Sintering of Bi(Ni0.5Ti0.5)O3-BiFeO3-Pb(Zr0.5Ti0.5)O3 Ceramics and Their Performance
Source: Materials (Basel). 2023 Apr 28;16(9):3459. doi: 10.3390/ma16093459 (PMC10180173; doi:10.3390/ma16093459)
Supplement: Supplementary file 1 [file materials-16-03459-s001.zip › materials-2332870-supplementary.pdf]

Supporting Information for

Low-Temperature Sintering of $Bi(Ni_{0.5}Ti_{0.5})O_3$-$BiFeO_3$-$Pb(Zr_{0.5}Ti_{0.5})O_3$ Ceramics and Their Performance

Wuyang Wang [1], Shihao Wang [2], Jun Sun [2], Qiushi Wang [2] and Bijun Fang [2,*]

[1] Bell Honors School, Nanjing University of Posts and Telecommunications, Nanjing 210023, China
[2] School of Materials Science and Engineering, Jiangsu Collaborative

Innovation Center of Photovolatic Science and Engineering, Jiangsu Province Cultivation Base for State Key Laboratory of Photovoltaic Science and Technology, National Experimental Demonstration Center for Materials Science and Engineering, Changzhou University, Changzhou 213164, China

* Correspondence: fangbj@cczu.edu.cn; Tel.: +86-519-86330095; Fax: +86-519-86330095

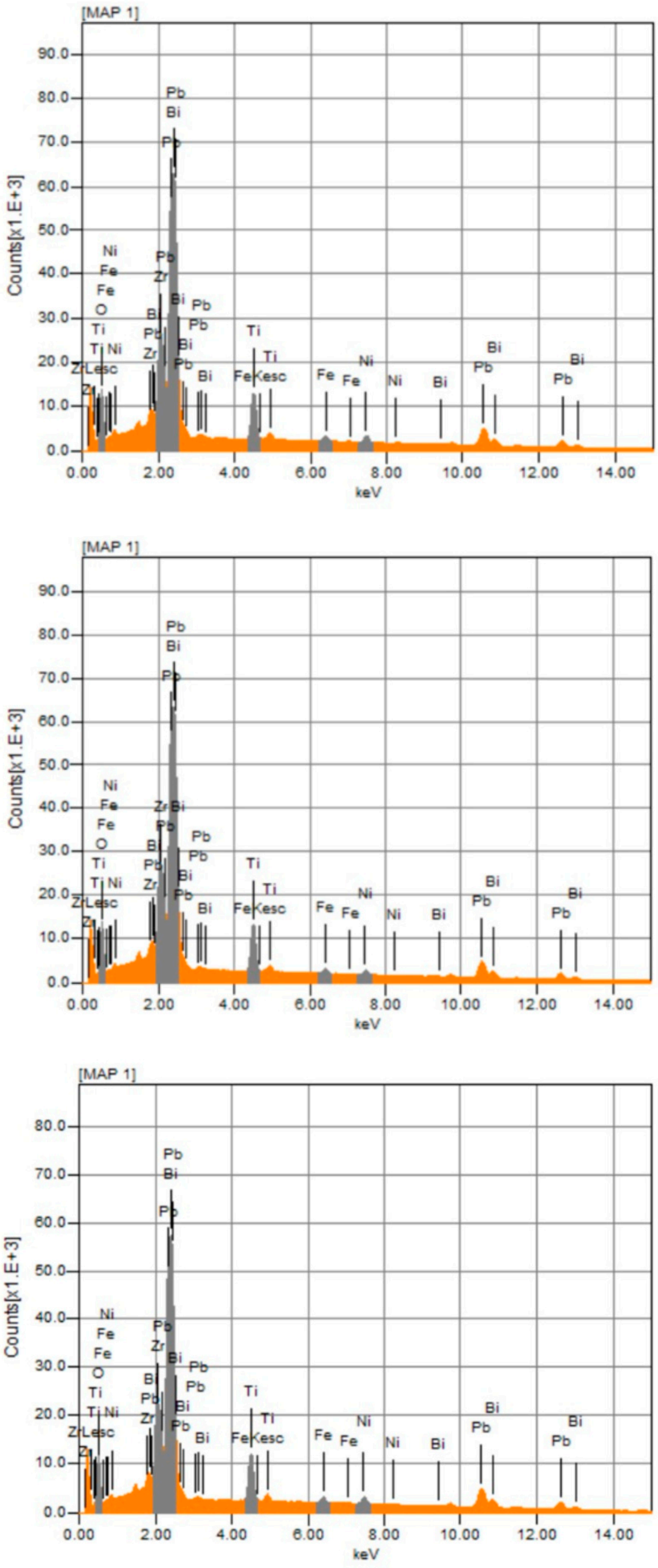

Figure S1. EDX spectra of 1030 ℃ sintered 0.21BNT-0.05BF-0.74PZT ceramics at different locations.

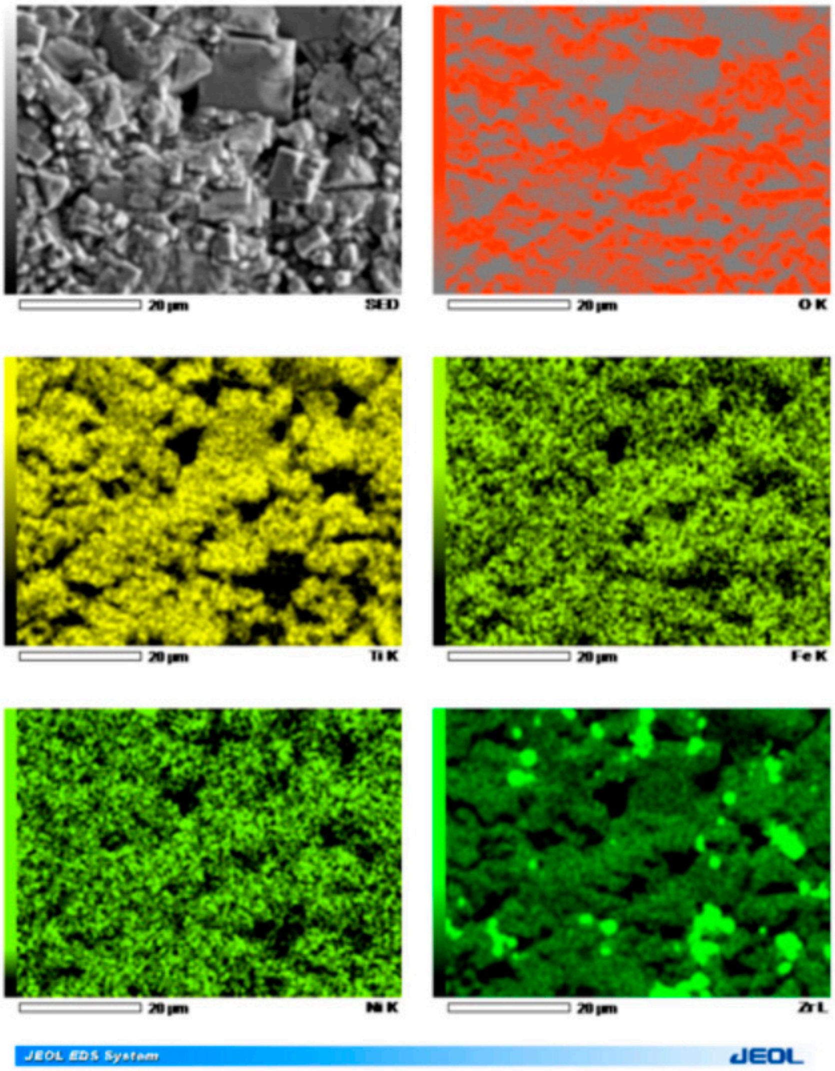
20 µm
SED
20 µm
O K
20 µm
Ti K
20 µm
Fe K
20 µm
Ni K
20 µm
Zr L
JEOL EDS System
JEOL

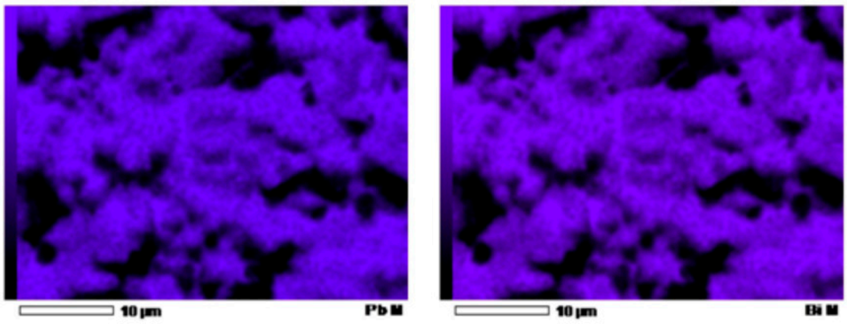
10 µm
Pb M
10 µm
Bi M

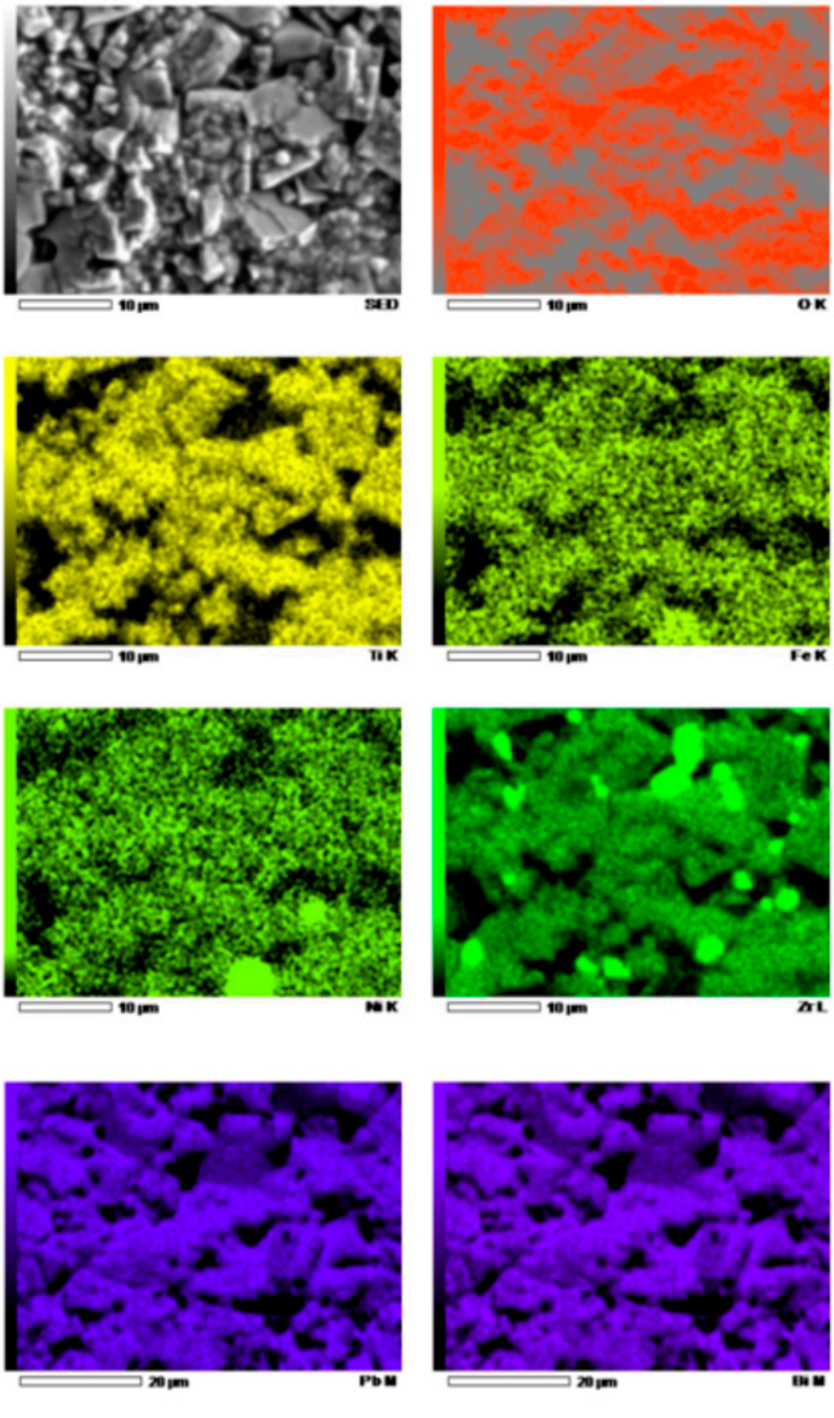
10 µm
SED
10 µm
O K
10 µm
Ti K
10 µm
Fe K
10 µm
Ni K
10 µm
Zr L
20 µm
Pb M
20 µm
Bi M

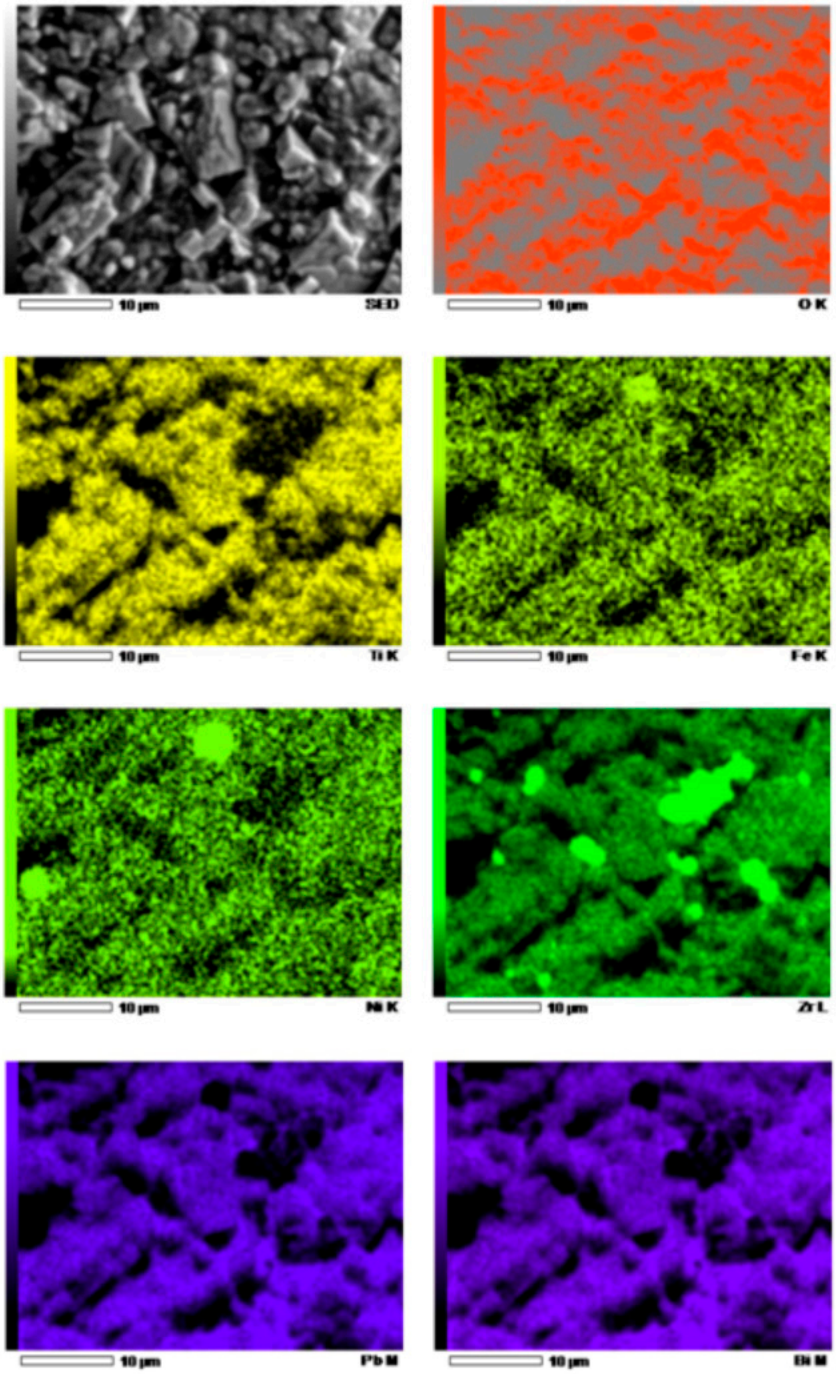

10 μm
SED
10 μm
O K
10 μm
Ti K
10 μm
Fe K
10 μm
Ni K
10 μm
Zr L
10 μm
Pb M
10 μm
Bi M

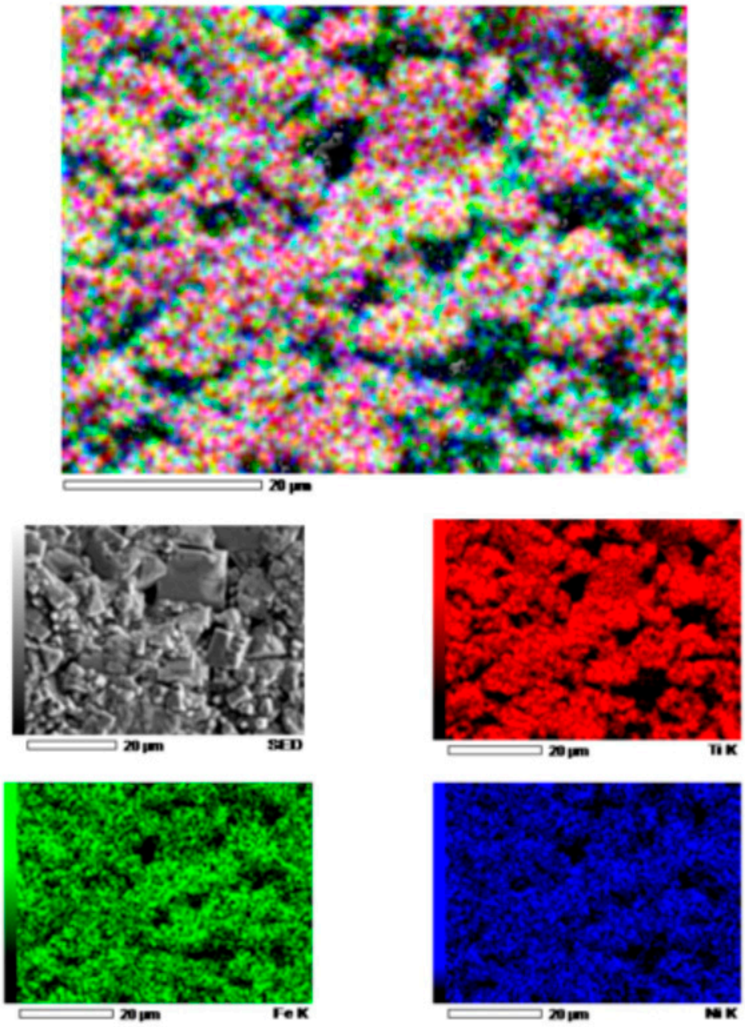

Figure S2. Element surface mapping of 1030 ℃ sintered 0.21BNT-0.05BF-0.74PZT ceramics at different locations.
